# Serum sCTLA-4 level is not associated with type 1 diabetes or the coexistence of autoantibodies in children and adolescent patients from the southern region of Saudi Arabia

**DOI:** 10.1186/s13317-020-00142-0

**Published:** 2020-12-03

**Authors:** Ahmed Al-Hakami

**Affiliations:** Department of Microbiology and Clinical Parasitology, College of Medicine, King Khalid University, P.O. Box 641, Abha, 61421 Saudi Arabia

**Keywords:** sCTLA-4, Type I diabetes (T1DM), Autoantibody coexistence

## Abstract

**Background:**

The soluble form of CTLA-4 (sCTLA-4) is associated with several autoimmune diseases. The aim of the study is to measure the serum sCTLA-4 levels in type I diabetic (T1DM) patients and to assess the presence of autoantibodies for a possible association.

**Methods:**

One hundred forty-two T1DM patients were enrolled in the study. Fifty of them were serologically positive for co-existing autoantibodies. One hundred and five subjects were enrolled in the study, as non-diabetic controls (1–17 years of age; median age—10 years). The serum samples of all the subjects were analyzed with ELISA to detect the concentration of sCTLA-4 and anti-GAD/IA2 IgG. Standard statistical analysis was conducted as required.

**Results:**

Ninety-four (66%) subjects of T1DM patients and five (4.7%) subjects of the non-diabetic group had antibodies positive for anti-GAD/IA2. Serum sCTLA-4 was low in most of the subjects of both the diabetic and control groups (p = 0.18). In the control group, nine individuals (8.6%) were positive for sCTLA-4. Similarly, only seven patients (4.9%) in the T1DM group had high levels of sCTLA-4, of which two were found to be double positive for anti-thyroid peroxidase and anti-thyroglobulin antibodies. In addition, among the T1DM patients, no significant relationships were observed between sCTLA-4 levels and age of onset (p = 0.43), disease duration (p = 0.09), or glycemic control (p = 0.32).

**Conclusion:**

Despite the previous findings of high sCTLA-4 levels in autoimmune diseases, serum levels of sCTLA-4 are not significantly different between T1DM patients and non-diabetic adolescents. Furthermore, we did not observe any association with autoantibody presence, glycemic control, or disease duration.

## Background

Type I diabetes (T1DM) is a chronic autoimmune condition characterized by progressive β-cell destruction and insufficient insulin production [1]. Similar to other autoimmune diseases, autoimmune reactivity in T1DM occurs as a result of multiple facets, including, genetic, environmental, and other aspects [2]. The presence of autoantibodies such as anti-islet antibodies, tyrosine phosphatase IA-2 (IA-2A), and glutamic acid decarboxylase autoantibody (GADA) is associated with the initiation of disease and are used to confirm T1DM diagnoses clinically [2]. The coexistence of autoimmune diseases with T1DM is well-known, especially for autoimmune thyroid disease and celiac disease [3]. Despite extensive genomic studies on genetic associations, only few genes have been strongly linked with T1DM. The identified genes include cytotoxic T lymphocyte antigen-4 (*CTLA-4*), which might also be associated with T1DM and many autoimmune diseases such as Graves’ disease, and autoimmune hypothyroidism [4].

Cytotoxic T lymphocyte antigen-4 (also known as CD152) is a glycoprotein receptor involved in the CTLA-4/B7 pathway, which controls the activation of T cells and acts as a checkpoint in peripheral T cell tolerance [5]. As opposed to CD28 molecules that activate T cells, CTLA-4 binds to CD80 and CD86 (B7-1 and B7-2) with high affinity and acts as a negative regulator [6]. It has been shown that *CTLA-4* knockout results in massive lymphocytic infiltration and the development of lymphoproliferative diseases; this demonstrated the major role of CTLA-4 in the control of autoimmunity and T cell activation [7]. Moreover, a soluble form of CTLA-4 (sCTLA-4) has been identified and might be associated with the regulation of T cell activation by CTLA-4 [8].

sCTLA-4 is normally present at low levels and is highly expressed in some autoimmune diseases [9, 10]. Studies have shown that sCTLA-4 is present in more than one autoimmune disease, including thyroid and autoimmune rheumatic diseases such as rheumatoid arthritis and ankylosing spondylitis [11–13]. A link between celiac disease (CD) and *CTLA-4* gene expression was found in 796 families with CD from six European countries [14]. Conversely, with T1DM patients, studies regarding *CTLA-4* gene expression are limited and conflicting, possibly because of differences in detection limits, sample sizes and genetic variations [15, 16].

Consequently, the *CTLA-4* polymorphism has been reported to be associated with several autoimmune diseases. It has been found that higher levels of sCTLA-4 and its associated receptor are expressed in patients with autoimmune diseases, which plays a key role in T cell regulation. However, a limited number of studies have measured sCTLA-4 levels in T1DM patient serum. Thus, the aim of the study is to assess sCTLA-4 expression levels in T1DM patients in southwestern Saudi Arabia. Further, the study will identify the expression levels of autoantibodies and whether the expression is similar as observed in other autoimmune diseases such as thyroid disease and CDs.

## Methods

### Patients and sample collection

Two-hundred and forty-seven subjects were enrolled in the study from January 2015 to January 2018. One hundred and forty-two of the subjects were T1DM patients who attended the diabetic center in Aseer Central Hospital for follow-up and 105 were non-diabetic subjects (either healthy volunteers or volunteers visiting the hospital for other non-inflammatory conditions such as trauma or non-urgent surgical patients). A questionnaire has been given to all the volunteers and those with allergies, asthma or associated inflammatory diseases are excluded. Blood samples (5 mL) from patients and healthy volunteers were collected in plain tubes and left for 30 min to clot at room temperature. Samples were centrifuged at 3500 RPM and serum was separated and stored at − 70 °C until use. All diabetic patients were tested for autoantibodies detected in CD (anti-tissue transglutaminase IgA and endomysial IgA) and thyroid disease [anti-thyroid peroxidase (TPO) and anti-thyroglobulin (TG)] as reported in the literature [17]. Twenty patients who were positive for celiac autoantibodies and 30 patients who were positive for thyroid autoantibodies were selected for further studies. Twenty-two patients were double positive, four were positive for anti-TPO only, and four were positive for anti-TG. All T1DM were enrolled randomly and no previous data was known about T1DM patients who showed autoantibodies positivity in the study (CD and thyroid) and no clinical data were retrieved from them.

All subjects were tested for anti-glutamic acid decarboxylase, tyrosine phosphate (GAD/IA2) and sCTLA-4. The anti-GAD/IA2 IgG pool was screened in serum samples with indirect ELISA using commercially available kits (Euroimmune, Lubek, Germany). Briefly, 50 µL of calibrators, positive controls, negative controls, and patient samples were added to wells and incubated overnight at 4 °C. The next day, plates were washed three times and 100 µL of GAD/IA2 biotin was added to the wells and incubated for 20 min at room temperature (RT; approximately 20–25 °C). Plates were washed three times and 100 µL of peroxidase-labeled avidin was added to the wells. The plates were incubated for 20 min at RT. Then, plates were washed and 100 µL of substrate solution was incubated in the wells for 20 min followed by the addition of 100 µL of stop solution. Plates were read at 405/620 nm, and IgG levels were obtained from a standard curve generated by an ELISA plate reader (Hmareader, Wiesbaden, Germany). Patients samples with a concentration ≥ 4 IU/mL were considered positive.

sCTLA-4 was measured in patient serum samples by sandwich ELISA using commercially available kits (BioOcean, Minnesota, USA). Briefly, 100 µL of each diluted standards or samples and 50 µL of detection antibody were added to assay plates and incubated with shaking for 3 h. After incubation, the plates were washed by six times, and 100 µL of substrate solution was added. The reaction was stopped by addition of 100 µL of stop solution, and plates were read at 450/620 nm using an ELISA plate reader. sCTLA-4 concentrations (pg/mL) in serum samples were obtained from a standard curve generated in the ELISA plate reader (Hmareader, Wiesbaden, Germany). Sensitivity of the sCTLA-4 ELISA kit is 0.71 pg/mL, intra-assay precision CV is 3%, inter-assay precision CV is 3.8%, and the assay range is 0.71–6000 pg/mL. Zero value indicates non-detectable level of serum sCTLA-4.

### Statistical analysis

Statistical comparisons of different variables were analyzed for significance using the statistical Package for Social Sciences (SPSS v.16). P values ≤ 0.05 were considered significant. Non-parametric Mann–Whitney U test or Independent t-tests were used to make comparisons between patient groups and different variables as required.

### Informed consent

Informed consent was obtained from all participants (n = 247).

### Ethical approval

The research related to human use complied with all relevant national regulations and institutional policies and were in accordance with the tenets of the Helsinki Declaration and approved by the Ethics Committee of the College of Medicine, King Khalid University (number REC # 2015-04-04).

## Results

247 subjects were enrolled in the study; 142 of them were patients with T1DM with an age ranging from 1.5 to 17.5 years (mean age is 8 years; median age is 12 years). Mean disease duration is 5.4 ± 3.2 and range is 0.5–13. The remaining, 105 with an age ranging from 1 to 17 years (mean age is 10.1 years; median age is 11 years) were non-diabetic (Table 2).

Among 142 individuals with T1DM, nighty-four (66%) were positive for GAD/IA2 IgG antibodies whereas only five non-diabetic subjects (4.7%) of 105 were positive for GAD/IA2 IgG antibodies. Twenty of the 142 T1DM patients were positive for celiac anti-tissue transglutaminase (atTG) and anti-endomysial antibodies (EMA) antibodies; and thirty were positive for thyroid (TPO and TG) antibodies (Table 1).

**Table 1:** T1DM patients and controls with detectable levels of sCTLA-4

	T1DM patients with detectable level of sCTLA-4							Control
	T1DM	T1DM						
		+ atTG	+ EMA	Double positive (atTG + EMA)	+ TPO	+ TG	Double positive (TPO + TG)	
Male	3	–	–	–	–	–	–	4
Female	4	–	–	–	–	–	–	5
Total	7	–	–	–	–	–	2	9

(–) = non-detectable level

**Fig. 1:**
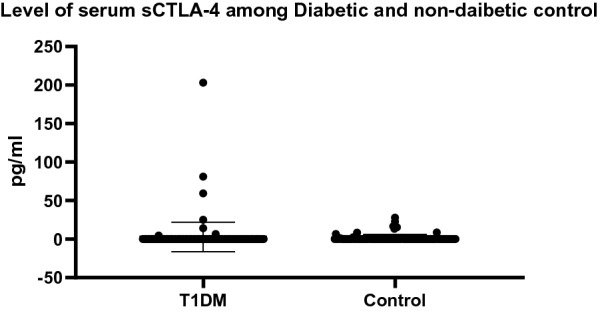
Levels of sCTLA-4 in T1DM patients and non-diabetic individuals (p = 0.18). *sCTLA-4* soluble form of cytotoxic T lymphocyte antigen-4, *T1DM* type I diabetes mellitus. *0 = under detection limit

It has been observed that sCTLA-4 was low in both the diabetic (mean ± SD 2.81 ± 19.17) and control groups (mean ± SD: 1.31 ± 4.67) without a significant difference (p = 0.18) (Fig. 1). Nine out of 105 in the non-diabetic control group showed detectable level of sCTLA-4 ranging from 0.9 to 27.9 (mean ± 13.8 ± 7.7) (Fig. 1). Out of the 142 in the T1DM group, only seven patients (4.9%) had detectable levels of sCTLA-4 ranging from 4.7 to 203 (mean ± SD: 56.3 ± 70.7). Further, out of these seven patients, two were positive for both anti-thyroid peroxidase and anti-thyroglobulin antibodies. In addition, serum sCTLA4 levels are not correlated with the autoantibodies. Finally, none of the 20 patients who tested positive for TG and EMA in the T1DM group showed detectable level for sCTLA-4 (Table 1).

All T1DM patients were reviewed for random blood glucose, HbA1c, duration, age of onset and family history to assess the possible relationship with serum sCTLA-4 concentrations. No significant correlations were observed between sCTLA-4 and these factors. Additionally, no correlations were found between anti-GAD/IA2 IgG antibody concentrations and sCTLA-4 or serum levels of thyroid antibodies (Table 2).

**Table 2:** Comparison of random blood sugar (RBS), Hb1Ac, duration, and age of onset between T1DM patients who has detectable or non-datable level of sCTLA-4

		Range	Median	Mean (± SD)	p value
Number	Detectable level of sCTLA-4 *n = 7				
	Non decetable *n = 135				
sCTLA-4 levels	Detectable	4.7–203	25.2	56.3	
	Undetectable	–	–	–	
RBS	Detectable	88–355	291.5	287.8 (± 27.6)	0.21
	Undetectable	62–481	293	251.3 (± 41.6)	
HbA1C	Detectable	6.9–16.6	9.6	9.6 (± 1.4)	0.32
	Undetectable	5–14	10	9.6 (± 2.1)	
Duration (years)	Detectable	0.5–13	5	5.4 (± 3.2)	0.09
	Undetectable	1–11	4	4.3 (± 2.4)	
Age of onset (years)	Detectable	1–16	7.5	8.7 (± 3.0)	0.43
	Undetectable	1–11	8	7.5 (± 3.7)	

*sCTLA-4* soluble form of cytotoxic T lymphocyte antigen-4, *T1DM* type I diabetes mellitus, *RBS* random blood glucose

## Discussion

Despite previous studies that reported increased sCTLA-4 serum concentrations in autoimmune diseases, the current study reports that sCTLA-4 expression is low in T1DM patients (only seven cases showed high levels of sCTLA-4 of 142 cases), with no significant difference compared to that in the non-diabetic control population (nine of 105 cases showed high level of sCTLA-4, p = 0.18). Thus, this study concludes there is no correlation between the level of sCTLA-4 and autoantibody presence with T1DM. T1DM is a result of multiple factors, including, genetic, environmental, and other triggering mechanisms [2]. In particular, genetic factors play an essential role in disease pathology. The specific incidence (one-tenth) is higher for the familial form, and the concurrence in monozygotic twins is higher than that in dizygotic and non-twin subjects [18]. Studies have identified the *CTLA-4* locus on chromosome 2q33 and its polymorphisms as one of 40 gene loci responsible for genetic susceptibility to T1DM [4, 19, 20]. Studies have shown that there is a significant correlation between *CTLA4* polymorphisms and T1DM prevalence in different ethnic populations such as Sudanese, Egyptian, Chinese, and Japanese [21–24]. Moreover, *CTLA-4* polymorphisms are associated with occurrence of the multiple autoimmune disorders [4], including type 1 diabetes.

In normal physiological conditions, two isoforms of *CTLA-4* mRNA are present, the full-length and soluble form [8]. The native soluble form was discovered 20 years ago, and has an immune regulatory function in mixed lymphocyte responses. However, its role as a marker of autoimmunity is contradictory and yet to be fully understood [11, 25]. This study has shown that the presence of serum sCTLA-4 is not varied in TIMD and in healthy patients. In contrast, several studies have shown high levels of sCTLA-4 in systemic lupus erythematosus, autoimmune thyroiditis and spondyloarthropathies [8, 25]. In a screening study, serum sCTLA-4 levels were higher in T1DM patients compared to healthy individuals among subjects younger than 15 years old [16]. However, in that study, serum sCTLA-4 levels were low in both T1DM patients and the control group (less than 3 ng/mL), whereas serum sCTLA-4 levels in patients with autoimmune thyroid disease ranged from 28 to 78 ng/mL [11]. Most studies that reported a correlation between the sCTLA-4 levels and autoimmune disease observed sCTLA-4 levels in the range of 10 ng/mL to more than 80 ng/mL. In contrast, to the study by Momin et al. [16], an in vitro study comparing activated PBMCs from 10 newly diagnosed T1DM patients, nine patients at the risk of developing T1DM, and 10 healthy individuals found that sCTLA-4 expression was significantly lower in T1DM patients [15]. A limitation of the latter study was the small sample size because the genetic and environmental factors play an essential role in disease pathology. In the current study among few patients sCTLA-4 levels were ranging from 4.7 to 203 pg/mL.

The coexistence of CD and autoimmune thyroid diseases with T1DM is well documented, and thyroid and celiac autoimmune disease prevalence can reach up to 10% and 30%, respectively, in patients with T1DM [3, 26]. Two thyroid antibodies that are considered indicators of autoimmune thyroiditis are anti-TPO and anti-TG. The principal antibodies used to diagnose CD are TG and EMA [3, 27]. The coexistence pattern of CD, autoimmune thyroid disease, and T1DM [3] in a cluster of patients indicates a possible common genetic background. A gene locus in chromosome 2q33, which contains *CTLA4* and *CD28*, is associated with the risk of T1DM, autoimmune thyroiditis and other autoimmune diseases [4, 19]. It has been shown that serum levels of sCTLA-4 were even higher in patients with autoimmune thyroiditis [11], spondyloarthropathies [13], ankylosing spondylitis [28], systemic lupus erythematosus [12], and CD [29] than in healthy individuals. Therefore, an investigation into the link between serum sCTLA-4 and autoimmune thyroiditis, CD and T1DM is warranted. In this study, we selected patients with both T1DM and CD or patients with T1DM and thyroid autoantibodies. Our results indicated that only seven T1DM patients had high sCTLA levels, and two of them were patients with T1DM and thyroid antibodies. No correlation was determined because only small group of TIMD patients (7 in number) have shown high sCTLA-4. Additionally, only two patients with thyroid antibodies had high sCTLA-4 levels. Furthermore, no T1DM patients with celiac autoantibodies had measurable sCTLA-4 levels. This is comparable to a study of the *CTLA-4* gene in celiac patients in the Basque Population that found no association between CTLA-4 and CD [30]. However, to rule out a possible link between CTLA-4 and the co-occurrence of autoimmune diseases and T1DM, further genetic studies are necessary.

CTLA-4 is a co-stimulator, binds B7, and acts as immune regulator of T cells upon expression. The function of serum sCTLA-4 is not well understood, and studies have reported contradictory findings. One study showed that sCTLA-4 has an immune regulatory effect, binds B7, inhibits CD86 function on T cells, decreases IFN-γ, IL-2, and IL-13 levels, and increases levels of TGF-β and IL-10 [8]. In support of a protective role for sCTLA-4, a knockdown experiment in mice showed that the downregulation of sCTLA-4 impairs CD25(+) CD4(+) regulatory T cell suppression and accelerates disease onset [31]. To determine the relationship between sCTLA-4 and disease duration or age of onset, we reviewed patient history and compared it with sCTLA-4 levels. No significant correlation was observed between disease duration, age of onset, or glycemic control and serum sCTLA-4 levels in the study population. Additionally, no significant correlation was found between serum sCTLA-4 levels and GAD/IA2 antibodies. Regarding the hypotheses that sCTLA-4 has a specific role in the early onset of the disease, experiments have shown that sCTLA-4 expression remains low in newly diagnosed T1DM patients and patients with T1DM 1 or 2 years after diagnosis [15]. Serum sCTLA-4 levels are typically low in healthy individuals and express at a higher level in patients with autoimmune diseases. Therefore, Pawlak et al. [32] suggested using serum sCTLA-4 as a marker for autoimmune diseases, and potentially as a therapeutic target.

Although HLA gene region is the major susceptibility loci implicated in T1DM, there is a lack of supporting research to consider sCTLA-4 as an indicator or predictor of T1DM. The *CTLA-4* gene encodes two major transcripts. One that encodes for trans-membrane protein in cytotoxic T cells that down regulates immune response. Another transcript encodes for a soluble immunoreactive protein, which lacks trans-membrane domain and is found in blood plasma. Berry et al. have tested four different SNPs within the CTLA-4 region in 54 patients with different autoimmune diseases and have found that there is no correlation of SNPs with the expression levels of sCTLA-4. This may be due to the haplotype nature of human genome among varied populations. However, the authors have found that the four SNPs contribute to the altered expression of sCTLA-4 mRNA and subsequently its protein expression [33–35]. Thus, our study is in agreement with the above studies and have shown that there is no difference in the expression of sCTLA-4 in both diabetic and control groups. To conclude, since a slight variation of the transcribed gene results in modified protein expression, very sensitive methods may be required to demonstrate the direct relationship of genotype with the protein expression, and function.

## Conclusions

In conclusion, our study demonstrated that serum sCTLA-4 does not correlate with T1DM in adolescents and is not associated with the coexistence of antibodies. No definitive relationship was identified between sCTLA-4 and disease duration and/or glycemic control. The future studies should include pre-diabetic patients as well as healthy subjects who are at high risk of developing T1DM to gain a clear insight on the role of circulating sCTLA4 in the pathogenesis and natural history of T1DM. Finally, it is also necessary to determine the function of *CTLA-4* genetic polymorphisms in T1DM patients.

## Data Availability

Material and data that support the findings of this study are available upon request to the corresponding author.

## Abbreviations

atTG: Anti-tissue transglutaminase
CD: Celiac disease
CTLA-4: Cytotoxic T lymphocyte antigen-4
EMA: Anti-endomysial antibodies
GADA: Glutamic acid decarboxylase autoantibody
IA2: Tyrosine phosphatase IA-2
sCTLA-4: Soluble form of CTLA-4
T1DM: Type I diabetes mellitus
TG: Anti-thyroglobulin
TPO: Anti-thyroid peroxidase
